# Range expansion of the invasive insect *Greenidea* (*Trichosiphon*) *psidii* (Hemiptera: Aphididae) in the Neotropical Region

**DOI:** 10.1186/s40064-016-2487-8

**Published:** 2016-06-16

**Authors:** M. P. Culik, J. A. Ventura, D. dos S. Martins

**Affiliations:** Instituto Capixaba de Pesquisa, Assistência Técnica e Extensão Rural CRDR-CN, Rodovia BR 101 Norte, Km 151, C.P. 62, Linhares, Espírito Santo CEP 29915-140 Brazil; Instituto Capixaba de Pesquisa, Assistência Técnica e Extensão Rural (Incaper), Rua Afonso Sarlo 160, Vitória, Espírito Santo CEP 29052-010 Brazil

**Keywords:** Biodiversity, Biogeography, Geographic distribution, Integrated pest management (IPM), Myrtaceae

## Abstract

*Greenidea psidii* is an invasive insect from Asia that feeds on a diverse variety of agriculturally and environmentally important plant species. As an essential component of research necessary for development of a better understanding of biodiversity and its conservation, this study documents a major recent expansion in range of *G. psidii* in the Neotropics to the region of the tropical restinga ecosystem of Brazil, where it was found infesting guava (*Psidium guajava*) and jabuticaba (*Plinia cauliflora*). A summary of information on the geographic distribution, host plants, identification, and potential natural enemies of *G. psidii* that may be useful for integrated management of this pest in the Neotropical Region and other areas where this invasive insect has recently become established and is likely to further spread is also provided.

## Background

The insect *Greenidea* (*Trichosiphum*) *psidii* van der Goot (Hemiptera: Aphididae) is an invasive pest that feeds on ecologically and economically important plants of the family Myrtaceae such as guava (*Psidium guajava*), and has been recorded from plants in a diverse variety of other families (Halbert 2004; Pérez Hidalgo et al. 2009). *Greenidea psidii* is originally from Asia and has recently invaded many other areas such as the United States, Costa Rica, and Mexico (Halbert 2004; Pérez Hidalgo et al. 2009; Salas-Araiza et al. 2011) where it has become established and has caused concern as a pest of agriculturally valuable crops such as guava as well as environmentally important native species such as jabuticaba (*Plinia cauliflora*). *G. psidii* was recorded for the first time in Brazil in 2004 on guava in the State of Paraná, and subsequently observed in Santa Catarina and São Paulo on guava, and on *Psidium cattleianum* in Paraná (Lazzari et al. 2006). This study reports a major new expansion in range of *G. psidii* in the Neotropics to the region of the tropical restinga ecosystem (Lacerda et al. 1984), where this aphid was found feeding on guava and jabuticaba.

## Results and discussion

Aphid specimens collected from a heavily infested guava and jabuticaba plants growing in Vitória, Espírito Santo in 2014 were identified as *G. psidii*. Collection data: *G. psidii*: Vitória, Espírito Santo, col. M. P. Culik, 23 July 2014, ex. guava (*P. guajava*); Vitória, Espírito Santo, col. A. do C. Carvalho, 20 November 2014, ex. jabuticaba (*P. cauliflora*).

*Greenidea psidii* is originally from Asia and in that region it has been recorded from Bangladesh, China, India, Indonesia (Java, Sumatra), Japan, Nepal, Pakistan, Philippines, and Taiwan (Halbert 2004). Recently, this invasive insect has spread to North, Central and South America and in these regions it has been found in the United States, in the States of California and Florida (Halbert 2004), in Brazil, in the States of Paraná, Santa Catarina, and São Paulo (Lazzari et al. 2006), Costa Rica (Pérez Hidalgo et al. 2009), Mexico (Salas-Araiza et al. 2011), and Panama and Venezuela (Cermeli et al. 2012). As the distance of the locale of this record of *G. psidii* in Espírito Santo is more than 700 km from the locale of previous records of the species in South America [Curitiba and Morretes, Paraná; Penha, Santa Catarina; and São Paulo, São Paulo, Brazil (Lazzari et al. 2006)], this record of *G. psidii* documents a major new expansion in the distribution of this invasive pest in the Neotropics. This is the first record of *G. psidii* in the region of the tropical restinga ecosystem, and thus, this invasive insect now represents an additional new threat to the native biodiversity of this ecosystem (Culik et al. 2013, 2014).

*Greenidea psidii* is an aphid which feeds on a diverse variety of plant hosts by sucking their sap which may weaken and damage such plants. Hosts of *G. psidii* include species in at least seven plant families including *P. guajava* and other Myrtaceae (species of *Callistemon, Eucalyptus, Eugenia, Melaleuca, Metrosideros, Plinia, Rhodomyrtus, Syzygium,* and *Tristania*), as well as species of *Glycosmis* (Rutaceae), *Engelhardtia* (Juglandaceae), *Ficus* (Moraceae), *Lagerstroemia* (Lythraceae), Rhamnus (Rhamnaceae) and *Scurrula* (Loranthaceae) (Halbert 2004; Pérez Hidalgo et al. 2009). As a plant feeding invasive pest *G. psidii* is especially of concern because it threatens agriculturally important crops such as guava as well as environmentally important native species such as jabuticaba.

Most members of the aphid subfamily Greenideinae, including *Greenidea*, have long siphunculi with correspondingly long setae (Halbert 2004) and therefore *G. psidii* can be distinguished from other aphid species common in Brazil based on easily observed characters such as the long siphunculi. However, the species *G. ficicola* Takahashi is also present in Brazil and *G. psidii* may be distinguished from *G. ficicola* as described by Halbert (2004): *G. ficicola* apterae have reticulations covering most of the length of the siphunculi, and alatae have 17–21 rhinaria on antennal segment III, in a line and not crowded or touching each other; whereas, *G. psidii* apterae have reticulations only at the base of the siphunculi and the siphunculi are ornamented with irregularly spaced spinules, and alatae have 20–31 rhinaria, some crowded and not in line with the others, and often touching. Additional information concerning the identification of aphid species is found in Blackman (2016).

As an invasive species it is likely that *G. psidii* has entered Brazil without its native natural enemies (Blossey 2011). Thus, introduction of host-specific natural enemies of *G. psidii* from its area of origin (classical biological control) may be useful for control of the aphid in areas where natural enemies of the pest are not currently present. Starý et al. (2010) provided information on four parasitoids that have been recorded from *G. psidii* in India and Bangladesh (Table 1) that may be useful for classical biological control of this insect in areas where its natural enemies are not present or not effective in preventing economically or environmentally damaging populations of the pest. In Mexico, Salas-Araiza et al. (2011) observed that three native *Chysoperla* (Neuroptera: Chrysopidae) predators fed readily on *G. psidii* and also noted nine species of coccinellids (Coleoptera: Coccinellidae) predators associated with the pest that may also contribute to control of this aphid (Table 1).

**Table 1:** Potential natural enemies of the invasive aphid *G. psidii*

Order: family *Species*	Location of observation^a^	Present in Brazil
Neuroptera: Chrysopidae		
*Chrysoperla carnea* (Stephens)	Mexico	No (de Freitas and Morales 2009)
*Chrysoperla comanche* (Banks)	Mexico	No (de Freitas and Morales 2009)
*Chrysoperla exotera* (Navás)	Mexico	No (de Freitas and Morales 2009)
Coleoptera: Coccinellidae		
*Coccinella* L. spp.	Mexico	Yes (CABI 2015)
*Cycloneda sanguinea* (L.)	Mexico	Yes (González 2012)
*Harmonia axyridis* (Pallas)	Mexico	Yes (González 2012)
*Hippodamia convergens* Guerin-Meneville	Mexico	Yes (González 2012)
*Hyperaspis quadrioculata* (Motschulsky)	Mexico	Not known
*Olla v*-*nigrum* Mulsant	Mexico	Yes (González 2012)
*Scymnus* Kugalann spp.	Mexico	Yes (González 2012)
*Stethorus* Weise spp.	Mexico	Yes (González 2012)
*Zoglobra* spp.	Mexico	Not known
Hymenoptera: Braconidae		
*Archaphidus greenideae* Stary & Schlinger	Bangladesh	No (Starý et al. 2010)
*Binodoxys greenideae* (Stary & Harten)	Bangladesh	No (Starý et al. 2010)
*Binodoxys trichosiphae* (Samanta & Raychaudhuri)	India	No (Starý et al. 2010)
*Lipolexis oregmae* (Gahan)	India	No (Starý et al. 2010)

^a^Reference source: Mexico, Salas-Araiza et al. (2011); Bangladesh, India, Starý et al. (2010)

## Conclusions

Increased knowledge of the occurrence and distribution of invasive pests such as *G. psidii* in the Neotropics is needed to inhibit dissemination of such pests to new areas in this region as well as to support research to reduce economically and environmentally harmful impacts of such organisms on crops and native biodiversity (Culik et al. 2013, 2014). Control of invasive insects such as *G. psidii* depends on integrated pest management (IPM) including biological control by native natural enemies of the pest if they are present, and introduction of host-specific natural enemies of the pest from its area of origin should be considered if native natural enemies are not effective in maintaining impacts of the pest below tolerable levels. Thus, additional research to determine the impacts and natural enemies of *G. psidii* in South America is needed to address potential negative economic and ecological impacts of this and similar invasive organisms in this region.

## Methods

A guava plant in Vitória, Espírito Santo, heavily infested with aphids was observed in July 2014 and samples of infested leaves of the plant were collected for identification of the insects. Approximately 100 adults and nymphs from several leaves were preserved in alcohol (Entomology Collection of Incaper, Vitória). Specimens were mounted on slides and identified as *G. psidii* based on Halbert (2004). In November 2014 specimens collected from a heavily infested, potted jabuticaba plant growing approximately 1 km from the original collection site in Vitória, Espírito Santo, were also identified as *G. psidii*.
